# Unmet need for critical care on the wards - how many critically ill patients are really out there?

**DOI:** 10.1186/2197-425X-3-S1-A470

**Published:** 2015-10-01

**Authors:** CP Newell, S Wallis, N Botting, C Sajdler, A Foo, C Bourdeaux

**Affiliations:** University Hospitals Bristol NHS Foundation Trust, Bristol, United Kingdom

## Introduction

The Bristol Royal Infirmary, UK, is a tertiary referral centre for cardiothoracic, upper gastro-intestinal and liver surgery, haematology and other services. The twenty bed intensive care unit (ICU) provides critical care services to some of these subspecialties as well as a general population of medical and surgical inpatients. There is no critical care outreach service provided by the intensive care unit so the study group aimed to evaluate whether there was an unknown population of critically ill patients being managed outside of the ICU who had not been discussed with, or referred to the ICU team.

## Objectives

**1.** To determine the burden of 'missed' potential critical care referrals as a proportion of the number of patients who were actually referred for critical care review.

**2.** To establish whether the inpatient population was more unwell, in terms of deranged physiology, during Winter, as is the perception [[1]].

## Methods

This study was undertaken on two separate days; one in August and one in December. All inpatient observation charts were reviewed for marked physiological variable derangement, with the exception of cardiac surgical, obstetric and paediatric patients as these were covered by other critical care teams. Haematology inpatients were also not included. Inclusion criteria were adapted from McQuillan *et al.* [[2]] and included any of:

- Early warning score (EWS) ≥4
- SpO_2_ ≤90% on 5L O_2_ or higher
- RR >30/min or < 8/min
- SBP < 90mmHg
- HR < 40 or >140/min
- GCS < 9 or U on AVPU

Patients were excluded if they had a do not attempt resuscitation order in place, or a ceiling of treatment decision below ICU level documented. All patients fulfilling the inclusion criteria were then reviewed by a senior intensive care registrar to determine whether they would benefit from admission to the ICU.

## Results

**Figure 1:**
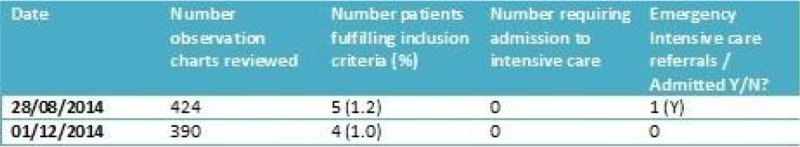
Figure 1

A total of 814 patient observation charts were reviewed over the two study days, the data is displayed in Figure 1. There was no significant difference in the percentage of patients with deranged physiology between the two dates; absolute difference 0.2% (p = 0.83). No patients fulfilling inclusion criteria required admission to the ICU.

## Conclusions

There was no significant difference in the number of unwell patients, based on markedly deranged physiological variables, being managed outside of the ICU between Summer and Winter on the study days analysed in our institution. Reviewing deranged physiology alone, there was no unmet need for critical care outside of the ICU; ward staff were effectively managing and referring patients when appropriate, without the presence of a critical care outreach service.
